# Validation of commercially available antibodies directed against subunits of the epithelial Na^+^ channel

**DOI:** 10.14814/phy2.15554

**Published:** 2023-01-12

**Authors:** Stephanie M. Mutchler, Shujie Shi, Sarah Christine M. Whelan, Thomas R. Kleyman

**Affiliations:** ^1^ Department of Medicine University of Pittsburgh Pittsburgh Pennsylvania USA; ^2^ Department of Cell Biology University of Pittsburgh Pittsburgh Pennsylvania USA; ^3^ Department of Pharmacology and Chemical Biology University of Pittsburgh Pittsburgh Pennsylvania USA

**Keywords:** antibody, ENaC, epithelial biology

## Abstract

The epithelial Na^+^ channel (ENaC) is traditionally composed of three subunits, although non‐canonical expression has been found in various tissues including the vasculature, brain, lung, and dendritic cells of the immune system. Studies of ENaC structure and function have largely relied on heterologous expression systems, often with epitope‐tagged channel subunits. Relevant in vivo physiological studies have used ENaC inhibitors, mice with global or tissue specific knockout of subunits, and anti‐ENaC subunit antibodies generated by investigators or by commercial sources. Availability of well‐characterized, specific antibodies is imperative as we move forward in understanding the role of ENaC in non‐epithelial tissues where expression, subunit organization, and electrophysiological characteristics may differ from epithelial tissues. We report that a commonly used commercial anti‐α subunit antibody recognizes an intense non‐specific band on mouse whole kidney and lung immunoblots, which migrates adjacent to a less intense, aldosterone‐induced full length α‐subunit. This antibody localizes to the basolateral membrane of aquaporin 2 negative cells in kidney medulla. We validated antibodies against the β‐ and γ‐subunits from the same commercial source. Our work illustrates the importance of validation studies when using popular, commercially available anti‐ENaC antibodies.

## 1 INTRODUCTION

The epithelial Na^+^ channel (ENaC) is a member of the ENaC/Degenerin superfamily of proteins that facilitates Na^+^ transport across high‐resistance epithelia in the kidney, lung, and colon. The channel plays a role in the regulation of blood pressure and fluid volume, with gain‐of‐function mutations leading to hypokalemia and hypertension (Grunder et al., 1997; Hansson et al., 1995; Shimkets et al., 1994). ENaC canonically consists of an α‐, β‐, and γ‐subunit with a δ‐subunit substituting for the α‐subunit in a tissue‐ and species‐specific manner (Canessa et al., 1994; Waldmann et al., 1995). More recently, ENaC expression has been shown in non‐epithelial tissues, including vascular smooth muscle, endothelium, and dendritic cells (Mutchler et al., 2021).

While each subunit contains a similar structure composed of two short intracellular termini connected to a large extracellular loop via two membrane‐spanning domains, the percent identity between the subunits varies between 23% and 34% (Hanukoglu & Hanukoglu, 2016). Areas of divergence within the subunits allow for differential interaction with regulatory‐specific factors including intracellular and extracellular [Na^+^], mechanical shear stress, acidic phospholipids, and posttranslational modifications including proteolytic cleavage, palmitoylation, and glycosylation (Kleyman et al., 2018; Kleyman & Eaton, 2020). Channel activity is also regulated by hormones such as angiotensin II, aldosterone, vasopressin, and endothelin (Nesterov et al., 2021; Rotin & Staub, 2021; Vendrov et al., 2022; Zaika et al., 2013).

Proteolytic cleavage is an important regulatory mechanism for ENaC, with short inhibitory sequences in both the α‐ and γ‐subunit requiring excision for full channel activity (Hughey et al., 2003, 2004). Whereas the α tract is cleaved twice by furin within the biosynthetic pathway, the γ‐subunit is only cleaved once by furin (Hughey et al., 2004). A second cleavage event that releases the γ tract is facilitated by a number of proteases at the membrane surface, as observed in the settings of aldosterone administration and volume depletion, as well as in cystic fibrosis and proteinuric kidney disease (Frindt et al., 2021; Hinrichs et al., 2019; Tan et al., 2014; Zachar et al., 2015).

ENaC activity has frequently been studied through patch clamp recording and Ussing chamber experiments. Mouse knockout models utilizing cre‐lox technology have also provided important physiological data in a cell type‐ or tissue‐ specific manner. With ENaC expression being reported beyond epithelia, the need for subunit‐specific antibodies has become increasingly important, as channel activity, subunit composition, and expression levels in non‐epithelial tissues vary widely. We aimed to validate commonly used commercial ENaC antibodies from StressMarq to ensure the integrity of our experimental results. We share here our findings regarding the validity of ENaC subunit‐specific antibodies and methodologies optimized for detection. Our results raise concerns with the anti‐α subunit antibody, as an intense non‐specific band appears on whole kidney immunoblots and a basolateral localization of the subunit was noted in immunostained kidney sections. The use of a previously characterized (Sorensen et al., 2013) anti‐α subunit antibody developed by the Loffing group along with results from epitope tagged ENaC expressed in Fisher rat thyroid cells confirmed the need to carefully evaluate results from experiments utilizing the Stressmarq anti‐α subunit antibody.

## 2 METHODS

### 2.1 Animal studies

All animal protocols conform to the National Institute of Health's Guide for the Care and Use of Laboratory Animals and were approved by the University of Pittsburgh IACUC. Male C57Bl/6 mice (Jackson Laboratories) were housed in a temperature‐controlled facility on a 12 h light/dark cycle. They were kept on standard laboratory chow, a 10% KCl diet (Envigo TD.09075) for 4 days, an 8% NaCl diet (Envigo TD.92012) for 4 weeks, or given aldosterone at a rate of 240 μg/kg/day via a subcutaneous minipump (DURECT Corporation, model 2002) for 2 weeks before sacrifice. Kidneys were collected and flash frozen or fixed in 4% paraformaldehyde for 24 h before being embedded in OCT.

### 2.2 Cell culture and overexpression of ENaC subunits

Fisher rat thyroid (FRT) cells were routinely cultured at 37°C and 5% CO_2_ in Gibco DMEM/F‐12 medium (Thermo Fisher, 11330‐032) supplemented with 8% FBS (Thermo Fisher, 10437‐028). Cells were seeded in 6‐well plates at 70% confluency and transiently transfected with mouse ENaC plasmids (0.5 μg per subunit) using Lipofectamine 3000 (Invitrogen, L3000008) according to the manufacturer's instruction. Only the α‐subunit had an N‐terminal HA epitope tag and a C‐terminal V5 epitope tag to generate trimeric channel complex of _HA_α_V5_βγ. Following 24–48 h incubation, transfected cells were lysed in 400 μl Goldstein buffer (20 mM HEPES, 100 mM NaCl, 40 mM KCl, 1 mM EDTA, 10% glycerol, 1% NP40, 0.4% deoxycholate, pH 7.4) supplemented with the protease inhibitor cocktail III (Calbiochem, 535140). Undissolved cell debris was removed by centrifuging at 10,000*g* for 10 min at 4°C.

### 2.3 Immunoprecipitation

Two percent of the FRT cell lysate was saved as inputs while the remaining lysates were split into three samples that were incubated with either 2 μg Stressmarq α antibody (SPC‐403) plus 40 μl rec‐protein G‐Sepharose (10‐1241, Invitrogen), only 40 μl rec‐protein G‐Sepharose (negative control) or 40 μl V5‐beads (positive control, S190‐119, Bethyl) overnight at 4°C on an end‐to‐end incubator. After extensive washes with PBS, 60 μl Laemmli sample buffer (1610737, BioRad Laboratories) was added to the beads and heated to 95°C to elute precipitated proteins. The whole cell lysates and IP samples were split and loaded into two identical gels, with one set of samples blotted with Stressmarq α antibody (0.5 μg/ml) and the other blotted with HA‐HRP (0.05 μg/ml, 3F10, Sigma) as a positive control.

To pulldown the α‐subunit in mouse tissues, 20 mg flash frozen lungs or kidneys of different treatments were homogenized in 500 μl Goldstein buffer and precleared with 50 μl protein‐G beads on ice for 45 min. Three percent of the whole lung lysate were saved as input and the rest were incubated with 2 μg Stressmarq α antibody (SPC‐403) and 40 μl Protein‐G beads on an end‐to‐end incubator overnight at 4°C. Immunoprecipitated samples were washed with PBS and eluted into 30 μl Laemmli sample buffer.

### 2.4 SDS‐page electrophoresis and immunoblotting

Flash frozen kidneys were homogenized in cold CelLytic lysis buffer (Sigma) supplemented with Halt protease and phosphatase inhibitor at 1:100 (Thermo Fisher). Homogenates were spun down at 4°C at 10,000*g* for 10 min. Protein was quantified using a BCA assay (Thermo Fisher). Samples were prepared in 4× Laemmli buffer (BioRad) and were either (1) left at room temperature for ~10 min, (2) put at 37°C for 30 min, or (3) heated at 97°C for 3 or 10 min. Samples were prepared with 40 μg of protein, unless otherwise noted, and run on 4%–15% Criterion TGX gels (BioRad) at 110 V for ~85 min. Gels were transferred using a wet transfer system at 400 mA for 35 min to a nitrocellulose membrane (BioRad). Membranes were blocked with 5% milk in PBS and were put in primary antibody (1 μg/ml) overnight at 4°C: anti‐α‐subunit (StressMarq, SPC‐403, lot #130911), anti‐β‐subunit (StressMarq, SPC‐404, lot #MK387434), or anti‐γ‐subunit (StressMarq, SPC‐405, lot #1112). Rabbit secondary HRP‐conjugated antibody (Jackson ImmunoResearch 711‐035‐152) was added to the blot for 1 h at room temperature. HRP‐conjugated secondary antibody that specifically recognizes light‐chain of rabbit IgG (LC‐only HRP, Jackson ImmunoResearch 211‐032‐171) was used in some experiments. Blots were developed using Clarity™ Western Blotting Substrate, followed by ClarityMax when needed (BioRad). Blots were imaged on a ChemiDoc™ Imaging System (BioRad). Blot quantification was performed using densitometry in ImageJ software, where necessary. Lot numbers represent antibodies utilized for the representative figures; however, consistent results have usually been noted across multiple lots. For comparison, an alpha subunit antibody kindly provided by Dr. Johannes Loffing was also utilized (Sorensen et al., 2013).

### 2.5 Immunofluorescent imaging

Tissue was fixed in 4% paraformaldehyde for 24 h at 4°C. After three washes in PBS for 10 min each, fixation was quenched by washing the tissue in 0.2 M ammonium chloride for 10 min. A PBS wash was again performed before the tissue was placed in 20% sucrose in PBS overnight at 4°C. Tissues were embedded in OCT and sectioned at 7 μm. Slides were placed in cold PBS containing 0.1% Triton‐X for 10 min followed by a PBS wash. Blocking was performed with a solution containing 0.6% fish skin gelatin and 10% horse serum at room temperature for 30 minutes. Primary antibodies were diluted in the same solution at the following concentrations: α‐subunit (10 μg/ml, StressMarq, SPC‐403), β‐subunit (10 μg/ml, StressMarq, SPC‐404), γ‐subunit (10 μg/ml, StressMarq, SPC‐405), and aquaporin 2 (AQP2) (1:200, Novus Biologicals, NBP1‐70378). Primary antibodies were left overnight at 4°C. After three washes in PBS, fluorescently conjugated secondaries were added for 1 h at room temperature, the slides were washed, and 4′,6‐diamidino‐2‐phenylindole (DAPI) was added to label nuclei. After one final wash, slides were dried and mounted with ProLong Gold Antifade Mountant (Thermo Fisher). Slides were imaged on a confocal microscope as stacks, presented as maximum projections.

## 3 RESULTS

### 3.1 Preparation of lysates at different temperatures

Each ENaC subunit antibody was initially tested with four different conditions to determine if temperature made a difference in the efficacy of detection of ENaC subunits. These conditions were tested in kidney tissue from mice on control diet that was flash frozen and run immediately after lysing (no freezing and thawing). The samples were prepared as one master mix that was aliquoted for the separate temperature treatments. The treatments were (1) 10 min at room temperature, (2) 30 min at 37°C, (3) 3 min at 97°C, or (4) 10 min at 97°C.

**FIGURE 1:**
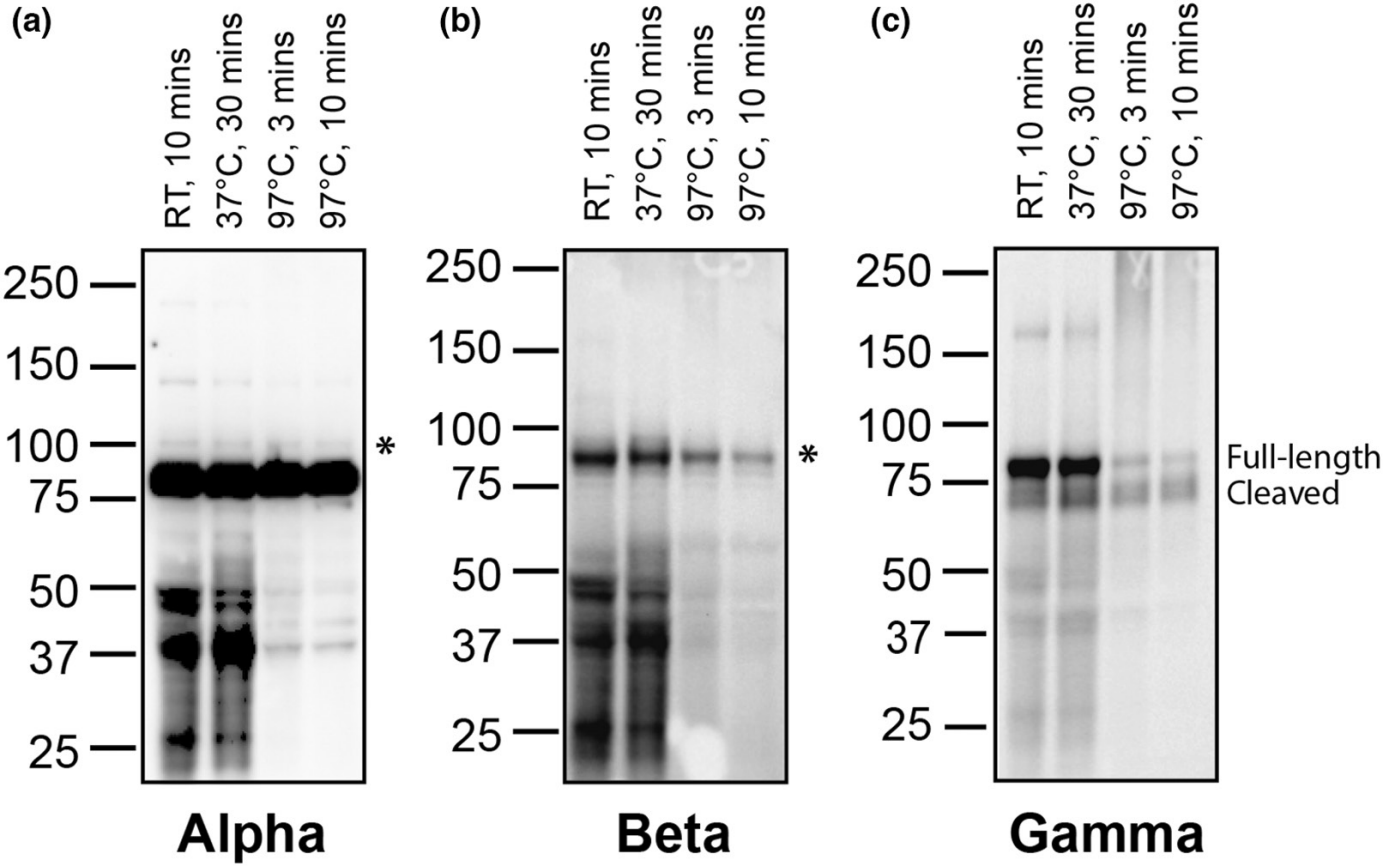
ENaC expression in kidney lysates prepared at different temperatures. Kidney homogenate was prepared in Laemmli buffer with beta‐mercaptoethanol either at room temperature (RT) for 10 min, 37°C for 30 min, or 97°C for 3 or 10 min (denoted for each lane). Three separate replicates of these conditions were run and the subsequent membranes were probed with Stressmarq antibodies directed against each ENaC subunit: (a) α, (b) β, or (c) γ. Asterisks denote the expected α‐ and β‐subunits on their respective membranes. The γ‐subunit displays both a full‐length band and a cleaved product. Blots are representative of results from at least three separate validations.

For both the α‐subunit and the β‐subunit immunoblots, the lower temperatures showed increased background bands in the lower half of the membrane as compared to the 97°C treated samples (Figure 1a,b). However, all temperatures showed a band corresponding to the correct size for the subunits, denoted by an asterisk. With the γ‐subunit, different temperatures altered the intensity of the full‐length γ‐subunit band in comparison to the cleaved γ‐subunit band (Figure 1c). The lower temperatures also caused less separation between the full‐length and cleaved bands, which could impact the ability to quantify these two populations separately.

### 3.2 Kidney but not lung shows difference in ENaC α‐ and γ‐subunit expression with aldosterone

Aldosterone regulates ENaC expression in the kidney by inducing the expression of the α‐subunit and increasing activating cleavage events in the γ‐subunit. This is tissue specific, as aldosterone does not have these effects in the lung, where expression is regulated by glucocorticoids (Renard et al., 1995). We utilized this differential regulation to test the antibodies in tissue homogenates to determine if we could observe well known trends in ENaC expression. Mice either were given a high salt (8% NaCl) diet to suppress aldosterone signaling or received exogenous aldosterone through a subcutaneous minipump to increase ENaC expression in the kidney. Both kidney and lung were collected, flash frozen, and homogenized. A total of 40 μg of protein per sample were added to 4× Laemmli buffer. Samples sat for at least 10 min at room temperature for the α‐subunit, they were put at 37°C for 30 min for the β‐subunit, and they were heated at 97°C for 3 min for the γ‐subunit.

**FIGURE 2:**
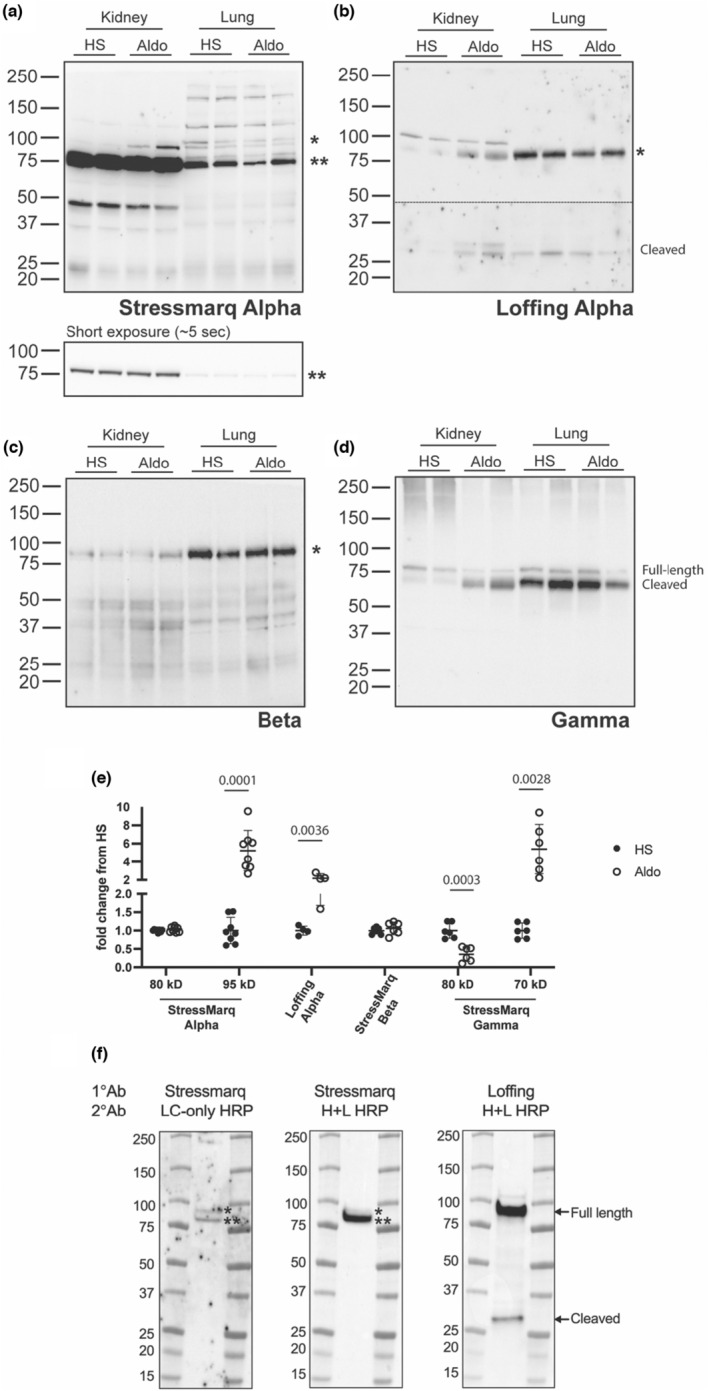
Mouse kidney, but not lung, demonstrate differences in ENaC α‐ and γ‐subunit expression with aldosterone. Lysate from mouse kidney and lung collected from animals on a high salt diet (HS) or with an aldosterone infusion (Aldo) were blotted for the presence of each ENaC subunit. (a) The Stressmarq anti‐α‐subunit antibody revealed an intense 80 kDa nonspecific band, denoted by ** and a 95 kDa full length α‐subunit, denoted by *. The top panel was exposed for a longer period (~7 min) to show the 95 kDa band and the bottom panel shows a quick initial exposure (~5 s) before saturation of the 80 kDa band occurred. (b) The blot was stripped and reprobed with a previously characterized antibody, produced in the Loffing laboratory. The panel shows two exposures, separated by a dashed line, to reveal both the full‐length 95 kDa α‐subunit, denoted by *, and a 30 kDa α‐subunit N‐terminal cleavage product. (c) The Stressmarq antibody directed against the β‐subunit shows the presence of a band at 90 kDa (denoted by *). (d) The Stressmarq antibody directed against the γ‐subunit revealed bands corresponding to a full‐length 80 kDa γ‐subunit and 70 kDa cleavage products, as indicated. (e) Signal from the kidney samples was quantified by densitometry, with each band normalized to total protein. Quantification is shown as a fold change from the average HS signal with *p* values shown for relationships that were significant (*p* < 0.05) as assessed by multiple *t*‐tests. (f) Mouse lung lysate was first probed with the Stressmarq α antibody, followed by a light chain only secondary antibody (LC‐only HRP). The blot was then stripped and reprobed again with the Stressmarq α antibody but followed by a whole IgG secondary antibody (H + L HRP). The blot was then stripped again and reprobed with the Loffing α antibody, followed by a whole IgG secondary antibody (H + L HRP). The Stressmarq antibody revealed an intense nonspecific band of ~80 kDa (denoted by **) and a full length α‐subunit migrating ~95 kDa (denoted by *). The Loffing antibody reveal both the full‐length 95 kDa α‐subunit and a 30 kDa N‐terminal α‐subunit cleavage product. Blots are representative of results from at least three separate experiments.

In the kidney, a band of high intensity immediately developed when probing for the α‐subunit (Figure 2a, denoted by **). However, this band had a calculated molecular mass of ~80 kD, which is smaller than that expected for the α‐subunit (Masilamani et al., 1999; Sorensen et al., 2013). Additionally, its intensity was similar across all samples, showing no difference in lysates from the high salt versus aldosterone treated kidneys (Figure 2e). Following a longer exposure, a single or doublet band was observed to migrate just above the 80 kDa band (Figure 2a, top panel). Importantly, this band (~95 kDa, denoted by *) was present in the aldosterone treated kidneys at a higher intensity than the high salt kidneys, showing its expression was induced by aldosterone (Figure 2e). In the lung, the doublet pattern was more pronounced, with expression not being dependent upon aldosterone (Figure 2a). The blot was stripped and reprobed with a well‐characterized α‐subunit antibody developed by Dr. Johannes Loffing's group to confirm the pattern of increased expression with aldosterone stimulation. This antibody demonstrated that expression of the 95 kDa α‐subunit is induced in aldosterone treated kidney (Figure 2e) whereas similar expression is maintained in the lungs of all mice regardless of treatment (Figure 2b, top). Additionally, it demonstrated increased levels of the ~30 kDa N‐terminal α‐subunit cleavage product in kidneys from mice treated with aldosterone as compared to those given high salt (Figure 2b, bottom). This cleavage product was absent from any blots probed with the Stressmarq α‐subunit antibody despite the fact both antibodies were produced against N‐terminal epitopes and should recognize this cleavage fragment. When detected using the Stressmarq antibody followed by a light‐chain only (LC‐only) secondary antibody, the intensity of the non‐specific band (denoted by **) was reduced, allowing longer exposure to detect the full length α‐subunit (denoted by *) in the lung lysate (Figure 2f). In addition, the molecular weight of the full length α‐subunit is consistent with the Loffing antibody, as demonstrated by stripping and reprobing of the same blot to allow for exact alignment (Figure 2f).

The β‐subunit appeared as a band at ~90 kDa (Figure 2c, denoted by *). Its expression in the kidney and the lung did not appreciably change with aldosterone treatment (Figure 2e). When probed for the γ‐subunit, the renal samples demonstrated that the subunit existed predominantly in its full‐length, uncleaved form of ~80 kDa (Figure 2d, top band) in the kidneys from high salt animals, while the administration of aldosterone caused cleavage of the subunit as demonstrated by an increase in the intensity of a faster migrating band of ~70 kDa (Figure 2e), representing C‐terminal cleavage fragments (Frindt et al., 2021; Masilamani et al., 1999). The lung tissue from all samples, regardless of treatment, exhibited a high level of cleavage, although the full‐length band was visible. This demonstrates that the antibody can distinguish between the cleaved and full‐length forms of the γ‐subunit.

### 3.3 Immunoprecipitation confirms the non‐specific α‐subunit band

**FIGURE 3:**
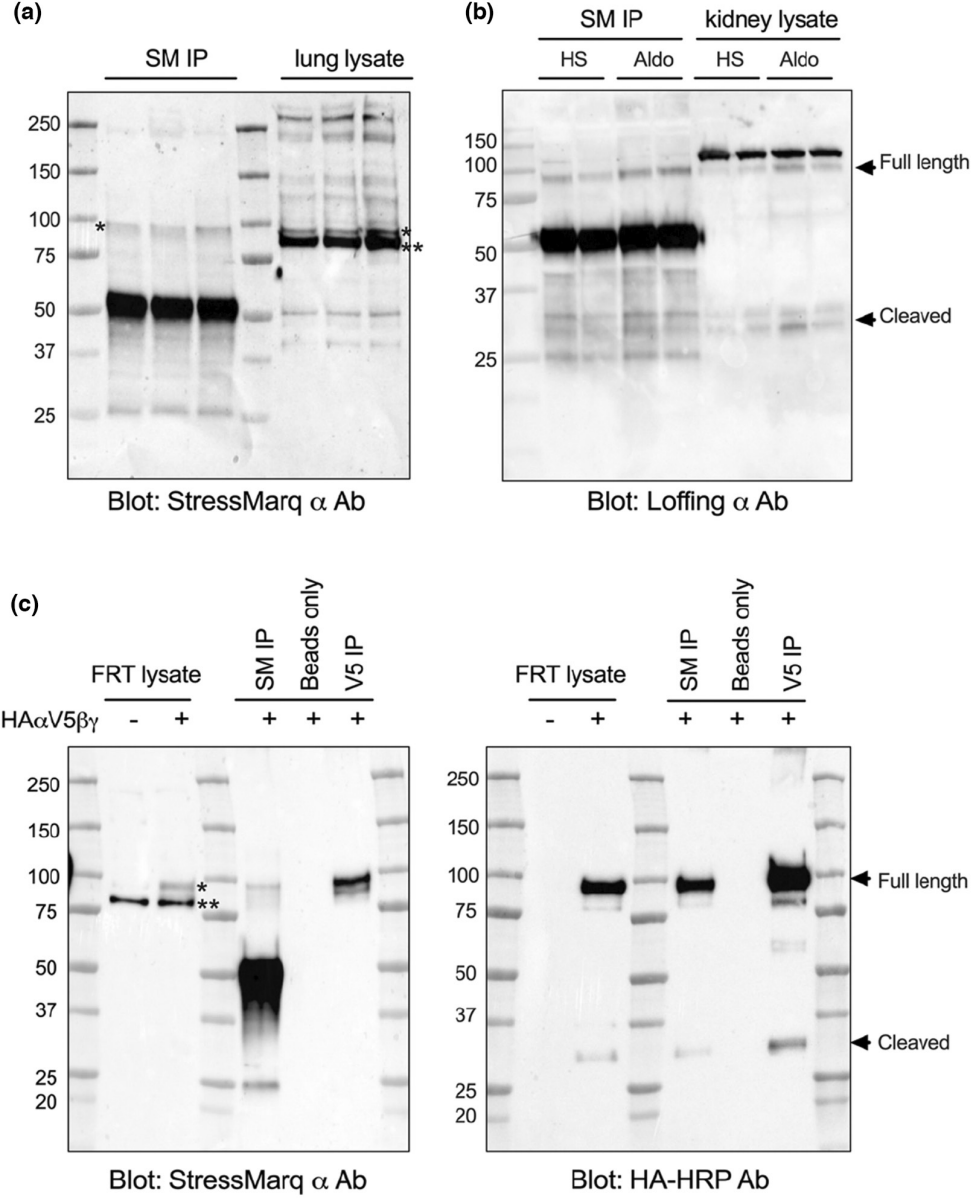
Immunoprecipitation eliminates the non‐specific α‐ subunit band in mouse lung tissue and FRT cells. (a) Mouse lungs were homogenized and incubated with the α‐subunit StressMarq antibody, followed by immunoprecipitation with protein G beads. Both the pulldown (left) and the lysate (right) were run together on a gel and blotted for the α‐subunit with the same StressMarq antibody as utilized for the IP. (b) Mouse kidneys collected from animals on a high salt diet (HS) or with an aldosterone infusion (Aldo) were immunoprecipitated with the StressMarq α antibody and protein G beads. Both the IP (left) and the kidney lysate (right) were run next to each other on an 8%–16% gel to probe for the α‐subunit with the Loffing α antibody. (c) Lysate from FRT cells either mock transfected or transfected with the three ENaC subunits were run for comparison (first two lanes). The lysate was incubated with either StressMarq (SM) anti‐α‐subunit antibody, beads alone, or V5‐tagged beads and subsequent pulldown was performed. The product was run on two separate gels simultaneously, with one being probed with the StressMarq anti‐α‐subunit antibody while the other was probed with an antibody directed against the HA tag. In all panels * illustrates the α‐subunit band while ** indicates to the non‐specific band. Results are representative of three separate experiments.

The presence of the high intensity 80 kDa band recognized by the StressMarq anti‐α‐subunit antibody in kidney lysates is likely an off‐target protein as the intensity of the 80 kDa band did not change with aldosterone treatment (Figure 2e) and it migrated faster than the full length α‐subunit recognized by the antibody raised by the Loffing laboratory (Figure 2b). To provide additional evidence that the 80 kDa band is an off‐target protein, immunoprecipitations of the α‐subunit were performed from both mouse tissues and FRT cells transfected with αβγ ENaC cDNAs where the α‐subunit had a C‐terminus V5 tag and an N‐terminus HA tag. Prior to immunoprecipitation, the 80 kDa band was present in both lung lysates and in mock transfected FRT cells when immunoblots were probed with the StressMarq anti‐α‐subunit antibody (Figure 3, denoted by **). Additionally, a slower‐migrating band (~95 kDa), representing the full length α‐subunit, was observed in lung lysates and in ENaC‐transfected FRT cell lysates (Figure 3, denoted by *) when blots were probed with the StressMarq anti‐α‐subunit antibody.

The StressMarq anti‐α‐subunit antibody and an anti‐V5 antibody were used for the FRT immunoprecipitation. The membrane was either blotted with the StressMarq anti‐α‐subunit antibody (Figure 3c, left) or an antibody against the HA tag (Figure 3c, right side). Both antibodies revealed the presence of bands at ~95 kDa, expected to be the full length α‐subunit. The anti‐HA antibody also revealed the 30 kDa cleavage product, regardless of the antibody utilized for pulldown, suggesting that the StressMarq α antibody was able to recognize and bind to both the full length and the cleaved α‐subunit during the immunoprecipitation, but it was not able to detect the cleaved product when used in blotting (Figure 3c). More importantly, immunoprecipitation of the α‐subunit with the StressMarq anti‐α‐subunit from mouse lung tissue followed by blotting with the same antibody eliminated the off‐target 80 kDa band (Figure 3a, denoted by **), while still recognizing the 95 kDa full length α‐subunit (denoted by *). ENaC α‐subunit was also pulled down from mouse kidneys using the StressMarq α antibody and blotted for with the validated antibody from the Loffing group. Both the full length and cleaved α‐subunit can be detected using the Loffing antibody in both total lysate and immunoprecipitated samples (Figure 3b), further confirming the specificity of the StressMarq α antibody for immunoprecipitation.

### 3.4 Linear quantification of ENaC subunits on immunoblot with serial dilution of lysate

**FIGURE 4:**
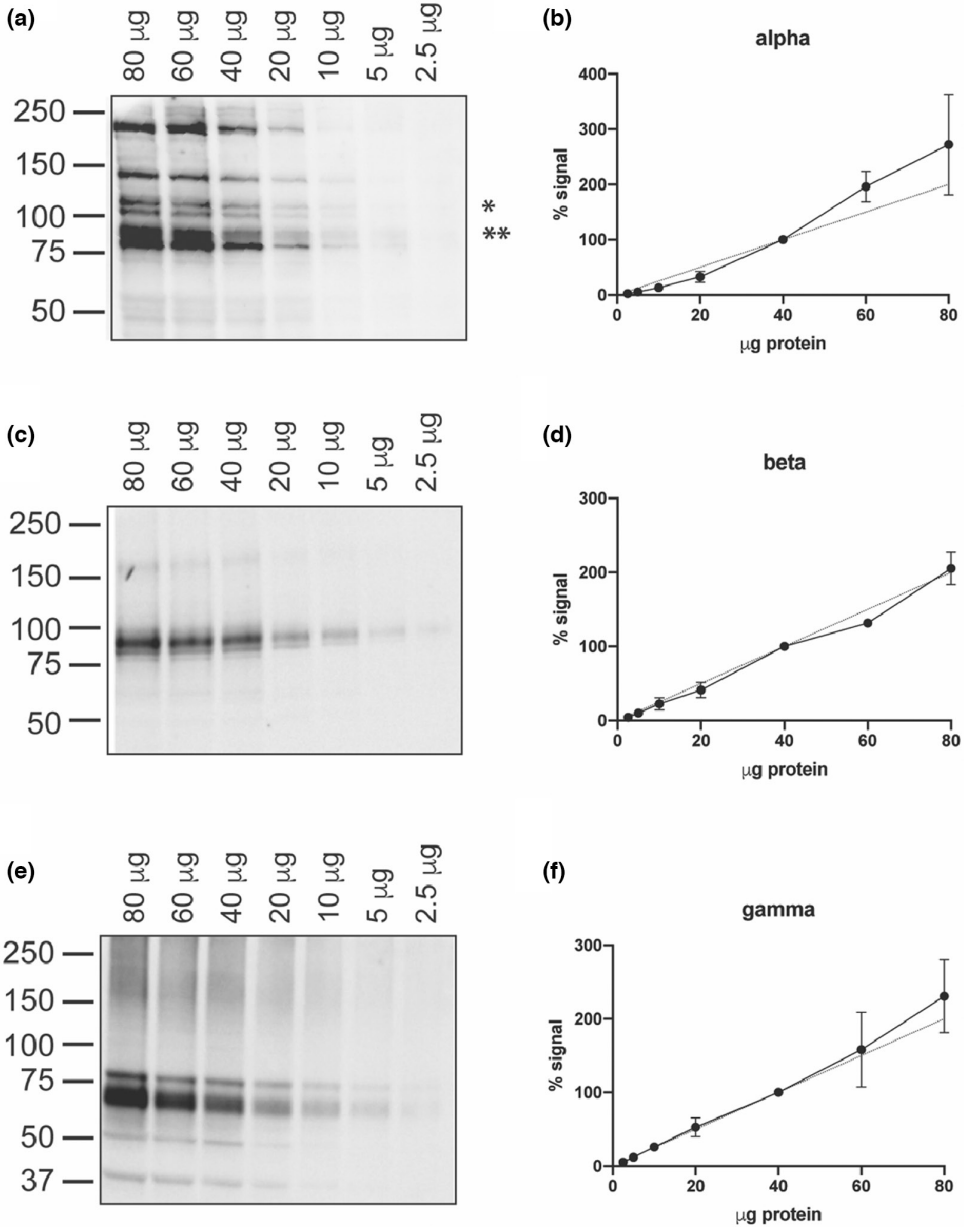
Subunit‐specific antibodies demonstrate linearity across a wide range of protein concentrations. Decreasing amounts of lung or kidney homogenate were probed for each subunit to determine the working range of the antibody. (a) Lung lysate ranging from 80 to 2.5 μg total protein, as denoted along the top of the blot, was probed for the α‐subunit using the StressMarq antibody. (b) The band of interest, denoted by *, was quantified and normalized to the value obtained for 40 μg (the halfway value) so that the results from three separate replicates could be combined. The dashed line demonstrates perfect linearity. (c) Kidney lysate was utilized for the β‐subunit and (e) the γ‐subunit. (d, f) The quantification of each band was again performed as described for (b), with both the full‐length and cleavage product bands being quantified for the γ‐subunit. Each graph represents results obtained from three separate experiments.

To detect differences in the level of protein expression between experimental samples, antibodies must have a large linear detection range. While this range can vary based on experimental methodology (i.e., method of detection, amount of protein loaded, etc.), knowing that an antibody can provide this working range is important. We tested whether the three ENaC subunit specific antibodies could provide this by performing serial dilutions of mouse tissue samples to test for linearity. Lung (α‐subunit) or kidney (β‐ and γ‐subunit) homogenates were first assessed by BCA to determine protein content and then prepared with 4× Laemmli buffer to achieve a mixture with 40 μg of protein per 12 μl. This was serially diluted 1:2 with buffer to achieve lower concentrations, while increasing amounts of the mixture were added per well for the two higher concentrations. When quantified, the density of the indicated bands plotted against the concentration of protein added per well showed a relatively linear range of detection for the α‐subunit between 2.5 to 80 μg of protein (Figure 4a,b), and a similar linear range of detection for the β‐subunit (Figure 4c,d) and γ‐subunit (Figure 4e,f).

### 3.5 ENaC subunit localization in mouse kidney

**FIGURE 5:**
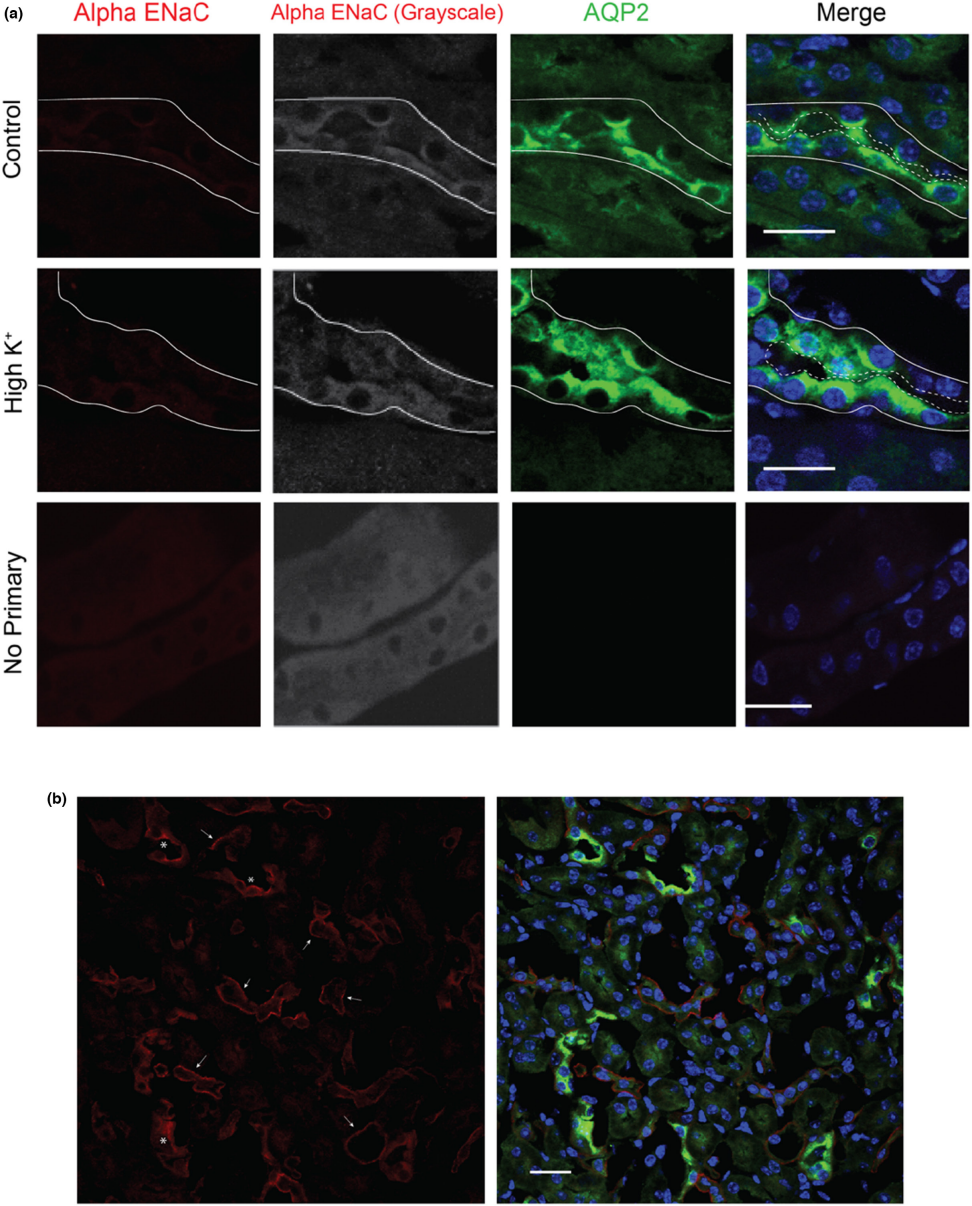
The α‐subunit antibody demonstrates minimal signal in AQP2‐positive cells and is mislocalized in kidney medulla. Kidney sections from mice kept on control diet (a, top) or 4 days of high K^+^ diet (a, middle) were labeled with the StressMarq anti‐α‐subunit antibody (red on left and converted to grayscale in second column). AQP2 (green) was used as a marker of the apical lumen of principal cells. The basolateral surfaces of tubules are denoted by solid lines and the apical surface by dashed lines. A no primary control is shown for comparison (a, bottom). (b) Positive staining for the α‐subunit (red) was only observed in the inner medulla of the kidneys, with representative images displayed here. While AQP2 positive cells within the medulla (green) did show expression of ENaC (denoted by *), the majority of the signal was localized to the basolateral side of both AQP2 positive and negative tubules (shown by arrows). Scale bar represents 20 μm in all images and images are representative of three separate regions examined in three mice of each treatment.

**FIGURE 6:**
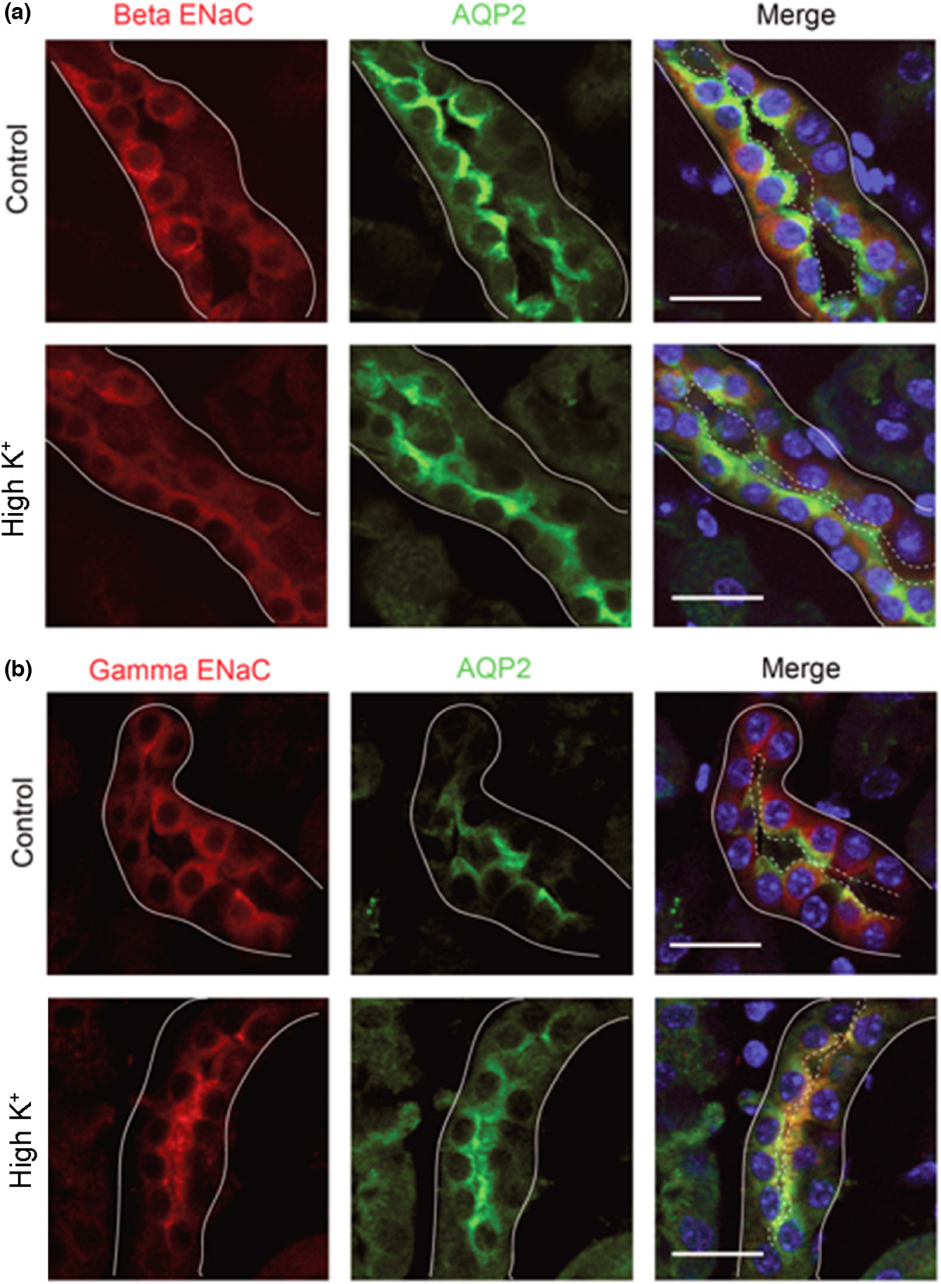
Immunofluorescent staining reveals movement of the β‐ and γ‐subunits from an intracellular location to apical surface with a high K^+^ diet. Kidneys from mice kept on a control diet (top) or 4 days of a high K^+^ diet (bottom) were probed for expression of (a) the β‐subunit of ENaC or (b) the γ‐subunit of ENaC. Sections were counterstained for AQP2 (green) to indicate principal cells. The basolateral surfaces of tubules are denoted by solid lines and the apical surface by dashed lines. Scale bars represent 20 μm and images are representative of three separate regions examined in three mice of each treatment.

We examined the localization of each ENaC subunit in mouse tissue to determine if the antibodies were sufficient for immunofluorescent imaging. Kidney tissue from mice that were unstimulated (controls) or that had been given a high K^+^ diet for 4 days to increase in aldosterone levels were stained for each of the subunits. An anti‐aquaporin‐2 (AQP2) antibody was utilized to denote principal cells (PCs). While it was expected that the α‐subunit would be present at low levels under control conditions, the antibody failed to demonstrate a strong signal in the apical membrane of PC in kidneys from mice on a high K^+^ diet. Instead, kidneys from both sets of mice showed no distinct apical α‐subunit signal in AQP2‐positive tubules (Figure 5a). Moreover, in kidneys from both conditions we noted a strong basolateral signal from cells in AQP2 negative tubules in the inner medulla (Figure 5b). This is not an expected localization of ENaC, and neither of the other two subunits showed this staining pattern (Figure 6), suggesting non‐specific labeling by the anti‐α‐subunit antibody.

For both the β‐subunit (Figure 6a) and the γ‐subunit (Figure 6b), localization was cytosolic in the control tissue, but localized more to the apical surface with high K^+^ diet, as assessed by its movement toward the AQP2‐labeled surface. This suggests the β and γ antibodies can be used to identify ENaC subunits with immunostaining, as both subunits are known to be constitutively expressed but trafficked to the surface with aldosterone stimulation (Masilamani et al., 1999).

## 4 DISCUSSION

The purpose of our study was to determine whether antibodies to the three ENaC subunits, commercially available through StressMarq, correctly identified these subunits by immunoblotting and immunofluorescent imaging in mouse tissues. Previously, work focused on the identification and quantification of the endogenous ENaC subunits had been challenging given the limited availability of well‐characterized commercial antibodies. However, even as more companies have developed subunit specific anti‐ENaC antibodies, characterization is often limited. In our hands, we found that anti‐β‐ and γ‐subunit antibodies from a commonly used commercial source, StressMarq, work well in identifying ENaC subunit expression with both immunoblotting and immunofluorescence imaging in murine tissues. However, we found an intense background band on immunoblots with the α‐subunit antibody that is likely a nonspecific band. Furthermore, the antibody gives an incorrect α‐subunit localization in mouse kidney with immunofluorescent staining. The correct full length α‐subunit is detected with this antibody on immunoblots, but the signal is quite low compared to the closely migrating non‐specific band. Additionally, the StressMarq anti‐α‐subunit antibody, raised against a synthetic peptide mapping to the amino acids 46–68 in the N‐terminus of the α‐subunit, does not detect the N‐terminal 30 kDa α‐subunit cleavage product. In contrast, the anti‐α‐subunit antibody produced by Loffing and colleagues raised against a sequence mapping to amino acids 2–21 within the N‐terminus, detects both the full‐length α‐subunit and the cleavage fragment.

We demonstrated that all three antibodies produce a signal in immunoblots with lysate treatments ranging from room temperature to 97°C (Figure 1a–c). However, these initial tests were done with fresh lysates that were never frozen and thawed. These conditions are not possible for many experiments where tissues are precious and where homogenates have been aliquoted and frozen for later use. In our experience, we have found that background bands near the β‐subunit increase after multiple freeze–thaws, making it difficult to determine the correct band for quantification. Additionally, the γ‐subunit bands become less defined, making separate analysis of the full‐length and cleaved protein more difficult. We have found the optimal conditions to prepare samples for SDS‐PAGE and prevent deterioration in quality after freeze–thaw are 30 minutes at 37°C for the β‐subunit and 3 min at 97°C for the γ‐subunit. Furthermore, our work and studies from other groups have shown that pretreating samples with N‐glycanase to remove N‐glycans enhances the ability to distinguish non‐cleaved, single‐cleaved, and double‐cleaved γ‐subunits (Frindt et al., 2021). Importantly, all samples must be treated equally within the same blot and when combining replicates across multiple blots, given the differences that occur in the ability to separate the full‐length and cleaved bands for the γ‐subunit. The temperature of the lysate can also affect the intensity of the full‐length band, demonstrating the need for stringent adherence to the same protocol.

Using the optimal conditions for each subunit, we demonstrated the large working range of all three antibodies (Figure 4). For a Western blot to be quantitative, the antibody must accurately reflect the differences in protein content across a wide range of protein level. We demonstrate here that all three antibodies were linear across a range of 2.5 μg up to at least 40 μg of total protein loaded per well, with a trend continuing up to 80 μg. While this may vary depending on the SDS‐PAGE gel composition, blotting membrane, specific HRP substrate, and the methodology used for signal detection, these results show the utility of these antibodies in being used for quantitation. However, each experimental setup, especially with different tissues or the use of an over‐expression system, will require validation of this linearity.

Our initial immunoblots all revealed the presence of a strong ~80 kDa band with the StressMarq anti‐α‐subunit antibody, which we suspected to be a non‐specific band given that its apparent molecular weight was lower than the predicted and the fact that it was not different between control kidney and aldosterone‐stimulated kidney tissue (Figure 2). In contrast, we readily detected aldosterone‐dependent differences in α‐subunit expression with the antibody generated by the Loffing group. This 80 kDa band was also present in mock‐transfected FRT cells. However, immunoprecipitating the α‐subunit from both lung and FRT cells transfected with ENaC eliminated the 80 kDa band, while maintaining the 95 kDa band (Figure 3). The presence of the non‐specific band has made quantification of the full‐length α‐subunit product challenging, given its close proximity and high intensity in comparison to the real band of interest. Using a light‐chain only secondary antibody may help separation by reducing the intensity of the non‐specific band recognized by the StressMarq anti‐α‐subunit antibody (Figure 2f). Immunofluorescent staining also proved problematic with the StressMarq anti‐α‐subunit antibody as no distinct apical signal was seen in AQP2 positive cells, while strong basolateral staining in the medulla was observed in AQP2 negative tubules (Figure 5). Given the well characterized expression of ENaC on the apical surface of AQP2 positive cells, the appearance of this strong basolateral staining suggested the antibody was detecting a non‐specific signal.

From these data, we are confident that this intense band seen with the StressMarq anti‐α‐subunit antibody is not the full length α‐subunit, but is a non‐specific protein. A number of manuscripts have been published with this anti‐α‐subunit antibody where immunoblots quantified this non‐specific band rather than the correct α‐subunit signal, given the size of the presented bands. If proper care is taken in running the gels for longer times to allow for greater separation of bands and the blots are given longer exposures or incubated with a specific LC‐only HRP secondary to reveal the correct α‐subunit band, we believe the antibody can be used to identify and quantify α‐subunit expression on immunoblots. However, the proper controls must be run, such as immunoblots of kidney lysates from mice on control diet versus kidney lysates from mice on a low Na^+^ or high K^+^ diet or following an aldosterone infusion. This antibody is not suitable to characterize α‐subunit expression via immunofluorescence in mouse tissue, especially in tissues where the expression pattern of ENaC is not well known, that is, tissue other than lung or kidney. For these experiments, other antibodies should be explored. If feasible, subunit‐specific knockout animals should be used to ensure staining is truly specific. As the Stressmarq α‐subunit antibody, directed against an N‐terminal epitope, does not detect the N‐terminal 30 kDa cleavage product, we encourage companies to raise antibodies against amino acids 2–21, the antigen sequence utilized by Loffing and colleagues.

## CONFLICTS OF INTEREST

The authors have no conflicts of interest to report.
